# Supplementary material for: IGF2BPs directly regulate the noncanonical translation of toxic proteins from mutant FMR1 mRNA containing expanded CGG repeats
Source: Nat Commun. 2025 Dec 10;17:569. doi: 10.1038/s41467-025-67261-y (PMC12808120; doi:10.1038/s41467-025-67261-y)
Supplement: Supplementary file 1 — Supplementary Information [file 41467_2025_67261_MOESM1_ESM.pdf]

IGF2BPs directly regulate the noncanonical translation of toxic proteins from mutant *FMR1* mRNA containing expanded CGG repeats

Anna Baud[1*], Damini Saha[2], Tomasz Skrzypczak[2], Izabela Broniarek[1], Daria Niewiadomska[1], Wojciech J. Szlachcic[1], Małgorzata Borowiak[1], Rajani Kanth Gudipati[2], Krzysztof Sobczak[1*]

[1]Department of Gene Expression, Institute of Molecular Biology and Biotechnology, Adam Mickiewicz University, Uniwersytetu Poznańskiego 6, 61-614 Poznan, Poland
[2]Center for Advanced Technologies, Adam Mickiewicz University, Uniwersytetu Poznańskiego 10, 61-614 Poznan, Poland

To whom correspondence should be addressed:

* Krzysztof Sobczak, Department of Gene Expression, Institute of Molecular Biology and Biotechnology, Adam Mickiewicz University, Uniwersytetu Poznańskiego 6, 61-614 Poznan, Poland, tel: +48 61 829 57 66
Email: ksobczak@amu.edu.pl
* Anna Baud, Department of Gene Expression, Institute of Molecular Biology and Biotechnology, Adam Mickiewicz University, Uniwersytetu Poznańskiego 6, 61-614 Poznan, Poland, tel: +48 61 829 59 52
Email: anna.baud@amu.edu.pl

Table of Content

1. Supplementary Figures
Supplementary Figure 1. Data related to Figure 1.
Supplementary Figure 2. Data related to Figure 2.
Supplementary Figure 3. Data related to Figure 3.
Supplementary Figure 4. Data related to Figure 4.
Supplementary Figure 5. Data related to Figure 5.
Supplementary Figure 6. Data related to Figure 6.
Supplementary Figure 7. Data related to Figure 7.

2. Supplementary Tables
a. Table S1. List of primers used in the study.
b. Table S2. List of siRNAs used in the study.

Supplementary Figure 1.

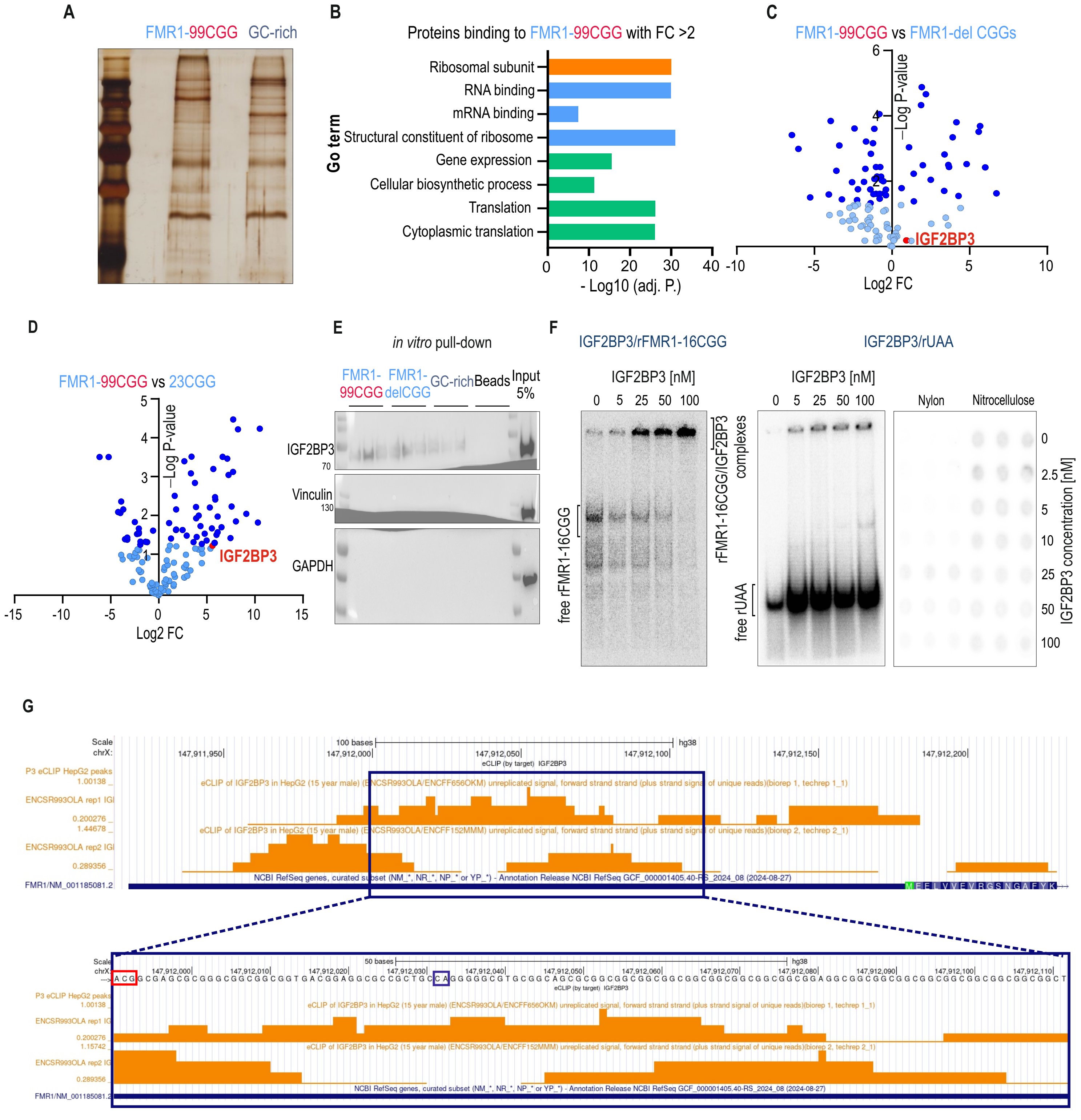

Figure S1. Data related to Figure 1. (A) SDS-PAGE of proteins eluted with FMR1-99CGG RNA and GC-rich RNA. Silver staining was performed to visualize protein bands. **(B)** Related to Fig. 1B: Gene Ontology (GO) analysis performed on 79 proteins interacting with rFMR1-99CGG, enriched FC >2 when comparing to GC-rich RNA. Graph presents significantly enriched, selected GO terms. **(C)** Enrichment of proteins interacting with FMR1-99CGG RNA compared to FMR1-delCGG RNA , and measured by label-free quantitative proteomics. Log2 fold-change and P values are given, indicating the magnitude of enrichment and statistical significance. IGF2BP3 protein (red dot) is not enriched on either of RNA baits. **(D)** Enrichment of proteins interacting with FMR1-99CGG RNA compared to 23CGG RNA , and measured by label-free quantitative proteomics. Log2 fold-change and P values are given, indicating the magnitude of enrichment and statistical significance. IGF2BP3 protein (red dot) is enriched on FMR1-99CGG RNA bait. **(E)** Related to Fig. 1C: Western blot of IGF2BP3 pulled down with in vitro transcribed biotinylated RNA molecules. Vinculin and GAPDH, which were not pulled down with RNA baits, are shown for comparison. **(F)** Related to Fig. 1D: Electrophoretic mobility shift assay and filter binding assay showing that recombinant IGF2BP3 binds in vitro with high affinity to ^{32}P-labeled FMR1-5'UTR RNA containing 16CGG repeats, but not to control $(UAA)_{20}$ RNA. (**G**) IGF2BP3 eCLIP reads coverage of 5'UTR of FMR1 transcript (upper panel) in HepG2 cells. Lower panel: zoom on the 5'UTR of FMR1 sequence showing ACG codon (red frame) and CA motif (purple frame). eCLIP data were downloaded form encodeproject and generated by Nostrand et al.[1]

Supplementary Figure 2.

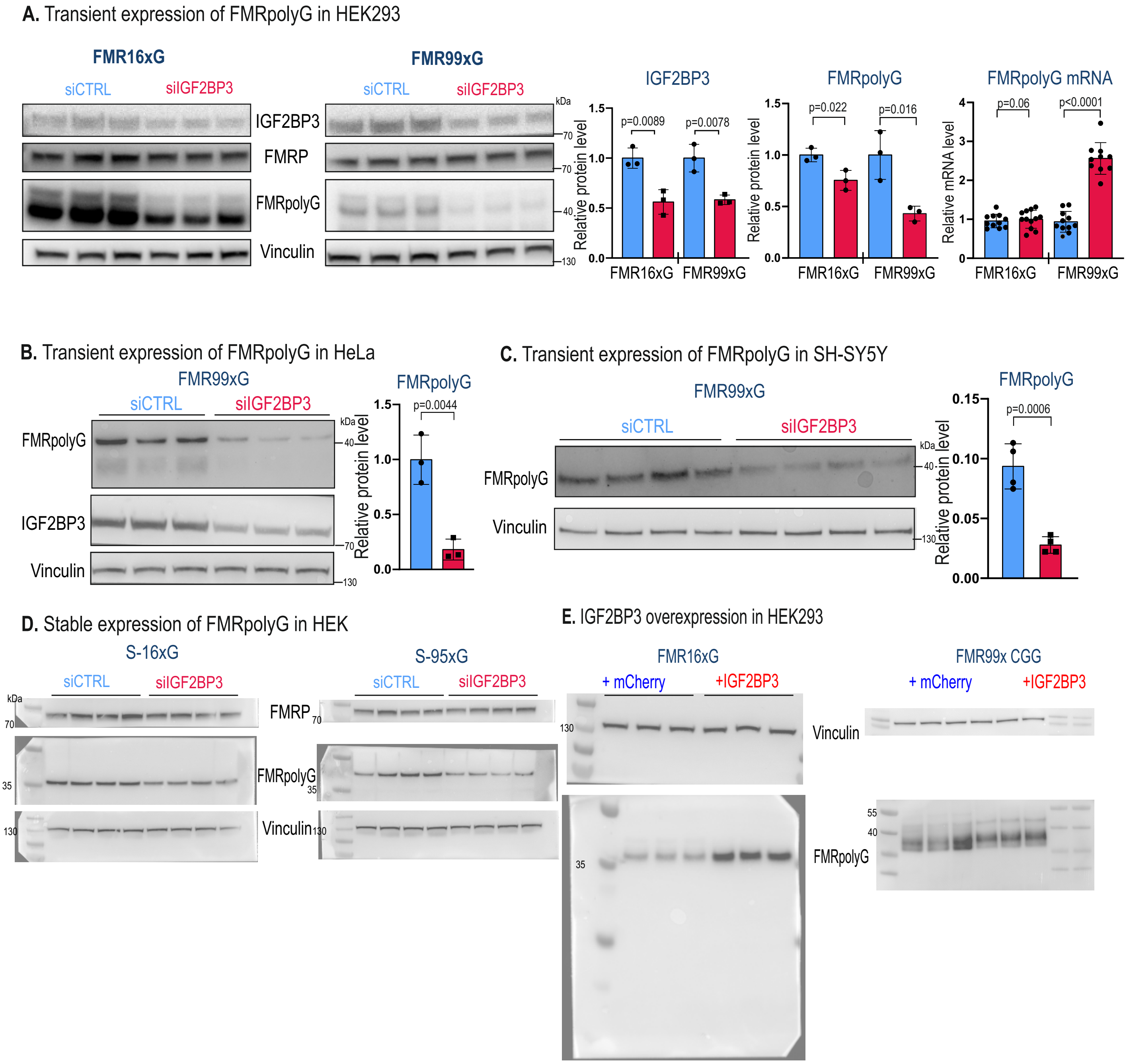

Figure S2. Data related to Figure 2. (A) Effect of IGF2BP3 KD (magenta) on RAN-translated FMRpolyG protein containing long polyglycine stretch (FMRx99G) or short polyglycine stretch (FMRx16G) and canonical IGF2BP3 and FMRP proteins in HEK293 cell model with transient FMRpolyG overexpression. Protein levels were measured by Western blot and normalized to Vinculin (left). Effect of IGF2BP3 silencing on *FMR16xG* and *FMR99xG* transgene expression quantified with RT-qPCR and normalized to *GAPDH* (right). The graphs present means from N = 3 biologically independent samples with SDs. **(B)** Effect of IGF2BP3 KD (magenta) on RAN-translated FMRpolyG protein containing long polyglycine stretch (FMRx99G) and IGF2BP3 protein in HeLa cells with transient FMRpolyG overexpression. Protein levels were measured by Western blot and normalized to Vinculin (left). Graph presents means from N = 3 biologically independent samples with SDs. **(C)** Effect of IGF2BP3 KD (magenta) on RAN-translated FMRpolyG protein containing long polyglycine stretch (FMRx99G) in neuroblastoma SH-SY5Y cells with transient FMRpolyG overexpression. Protein levels were measured by Western blot and normalized to Vinculin (left). Graph presents means from N = 3 biologically independent samples with SDs. **(D)** *Related to Fig. 2A.* Western blot used for quantification of FMRpolyG levels in Fig. 2A. **(E)** *Related to Fig. 2C.* Western blot used for quantification of FMRpolyG levels in Fig. 2C. (**A-C**) An unpaired two-tailed t-test was used to calculate statistical significance: ns, non-significant.

Supplementary Figure 3.

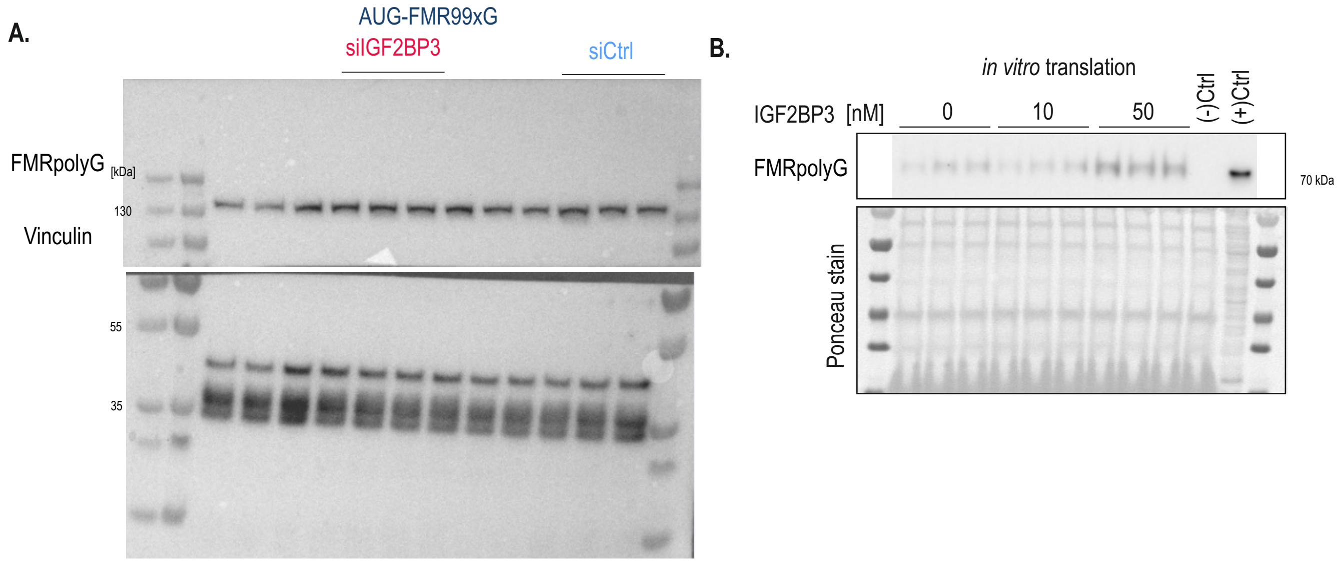

C.

mut16xG-nLuc

```
CTCAGTCAGGCGCTCAGCTCCGTTTCGGTTTCACTTCCGGTGGAGGGCCGCCTCTGAGCGGGCGGCGG
GCCGACGGCGAGCGCGGGCGGCGGCGGTGACGGAGGCGCCGCTGCTGGGGGGCGTGCGGCAGCGCGGC
GGCGGCGGCGGCGGCGGCGGCGGCGGCGGCGGCGGCGGCGGCTGGGCCTCGAGCGCCCGCAGCCC
ACCTCTCGGGGGCGGGCTCCCGGCGCTAGCAGGGCTGAAGAGAAGATGGAGGAGCTGGTGGTGGAAGT
GCGGGGCTCCAAAAGCGCTTTCTACAAGGGGGGTCTTCACACTCGAAGATTTCGTTG...
```

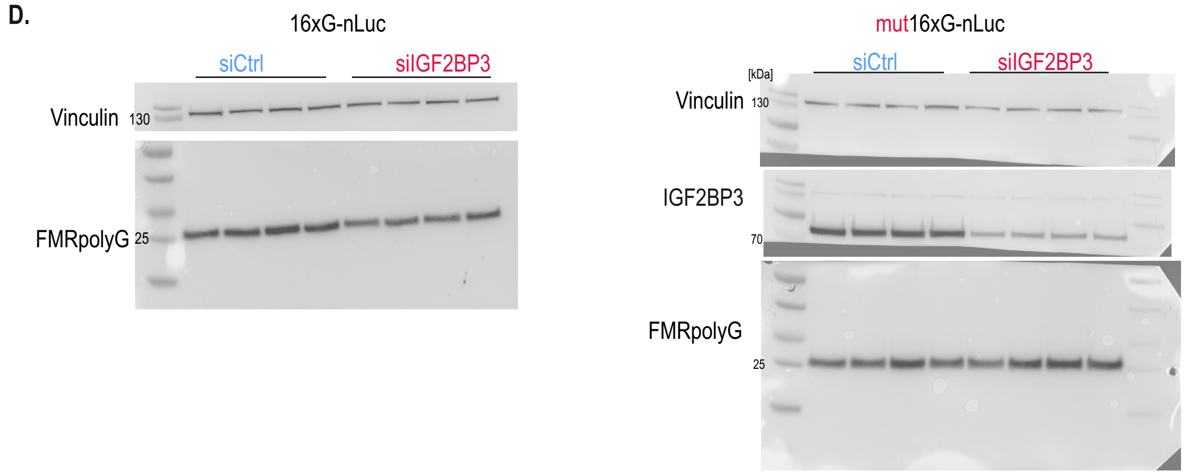

Figure S3. Data related to Figure 3. (A) *Related to Fig. 3A.* Western blot used for quantification of FMRpolyG levels in Fig. 3A. **(B)** *Related to Fig. 3B.* Western blot used for quantification of FMRpolyG levels in Fig. 3B, shown together with Ponceau stain of the membrane. **(C)** *Related to Fig.* 3C. Sequence fragment of mut16xG-nLuc construct. *FMR1* 5'UTR sequence is shown in black, FMRpolyG ORF is shown in bold, ACG initiation site is shown in green, mutated CA -> TG motif is shown in red, fragment of nanoLuc sequence is shown in magenta. **(D)** *Related to Fig. 3C.* Western blot used for quantification of FMRpolyG levels in Fig. 3C.

Supplementary Figure 4.

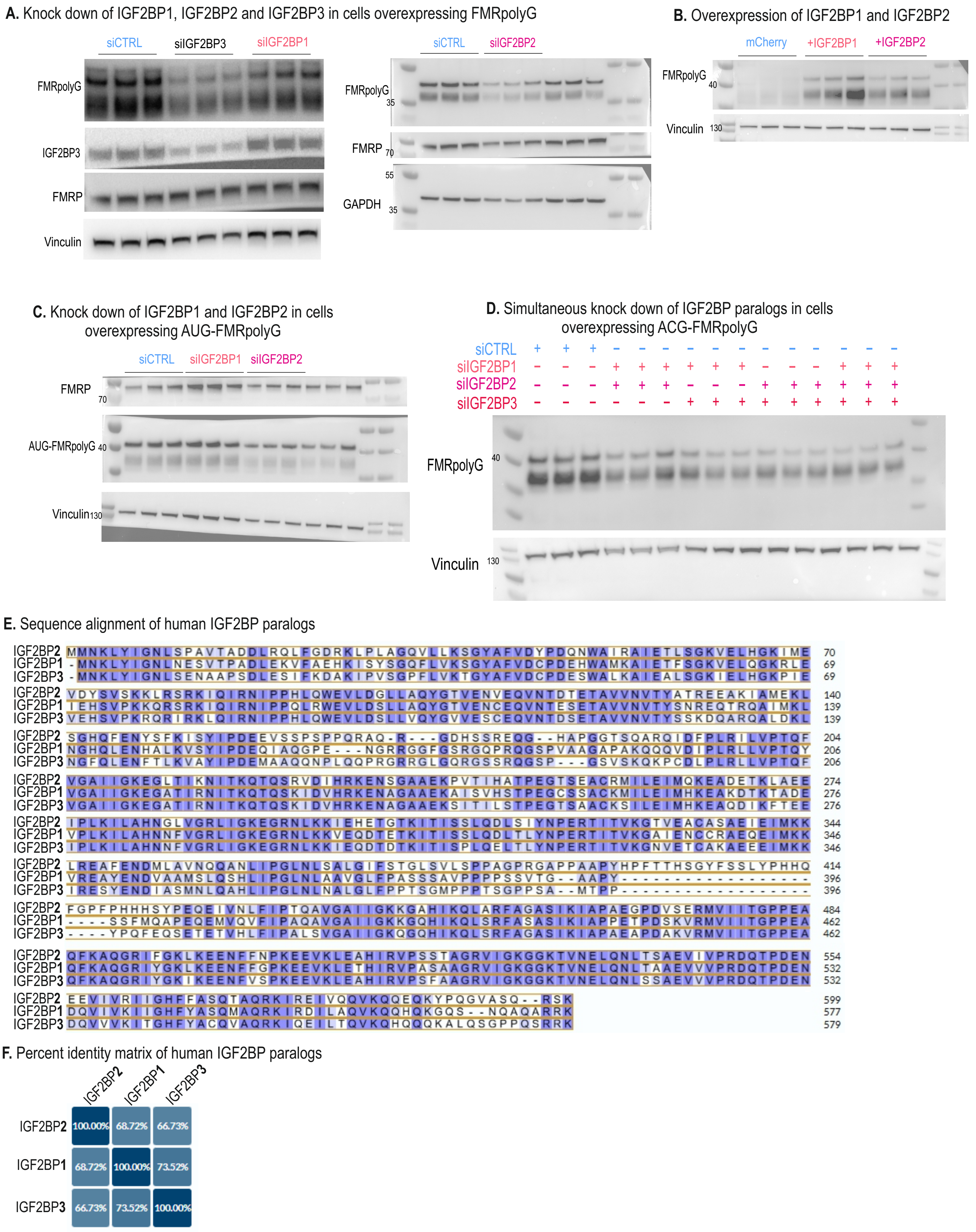

Figure S4. Data related to Figure 4. (A) *Related to Fig. 4A.* Western blots used for quantification of FMRpolyG levels in Fig. 4A. **(B)** *Related to Fig. 4B.* Western blots used for quantification of FMRpolyG levels in Fig. 4B. **(C)** *Related to Fig. 4C.* Western blots used for quantification of FMRpolyG levels in Fig. 4C. **(D)** *Related to Fig.4E.* Western blots used for quantification of FMRpolyG levels in Fig. 4C. **(E)** Clustal 0 (1.2.4) multiple amino acid sequence alignment of human IGF2BP paralogs. Sequence similarity among the paralogs is highlighted in blue. **(F)** Percent of amino acid sequence identity between IGF2BP paralogs. IGF2BP1 and IGF2BP3 share 73% of amino acid sequence identity.

Supplementary Figure 5.

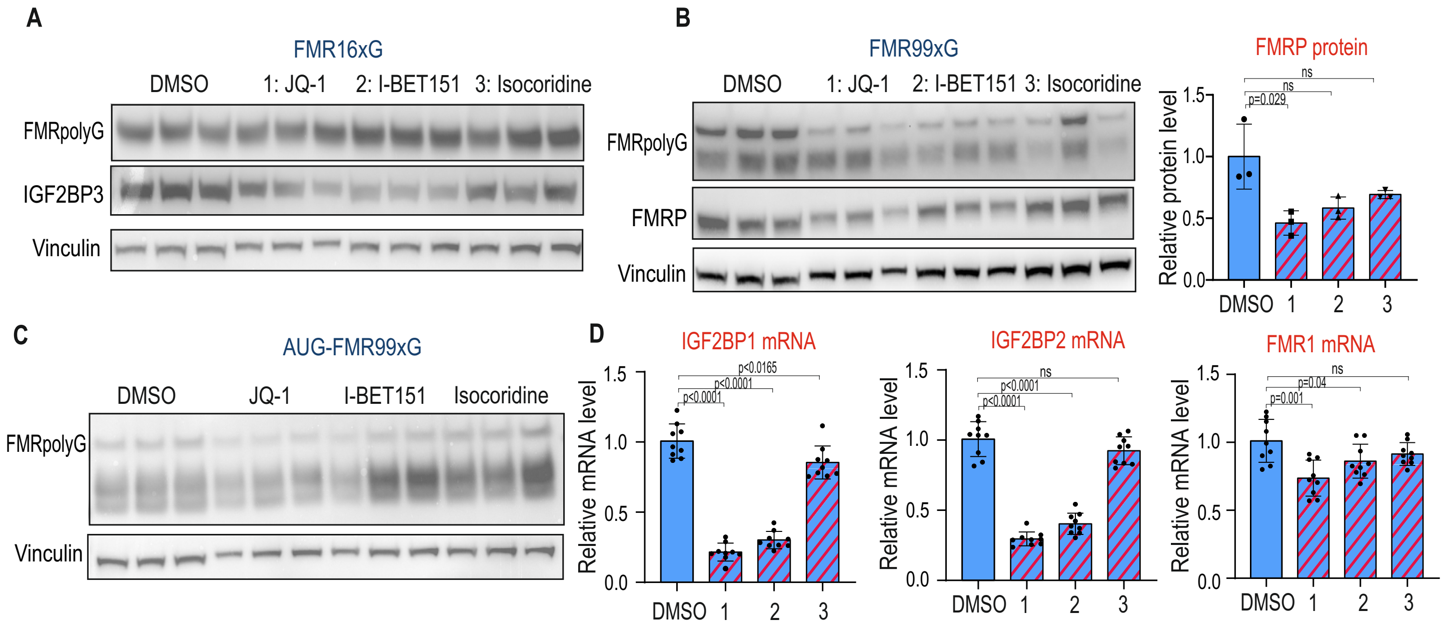

Figure S5. Data related to Figure 5. (A) Related to Fig. 5A. Western blots used for quantification of FMRpolyG levels in Fig. 5A. **(B)** Related to Fig. 5B. Western blots used for quantification of FMRpolyG levels in Fig. 5B. **(C)** Related to Fig. 5C. Western blots used for quantification of FMRpolyG levels in Fig. 5C. **(D)** The effect of JQ-1 (1), I-BET151 (2), and Isocoridine (3) on endogenous *IGF2BP1*, *IGF2BP2* and *FMR1* mRNA levels measured by RT-qPCR and normalized to *GAPDH*.

Supplementary Figure 6.

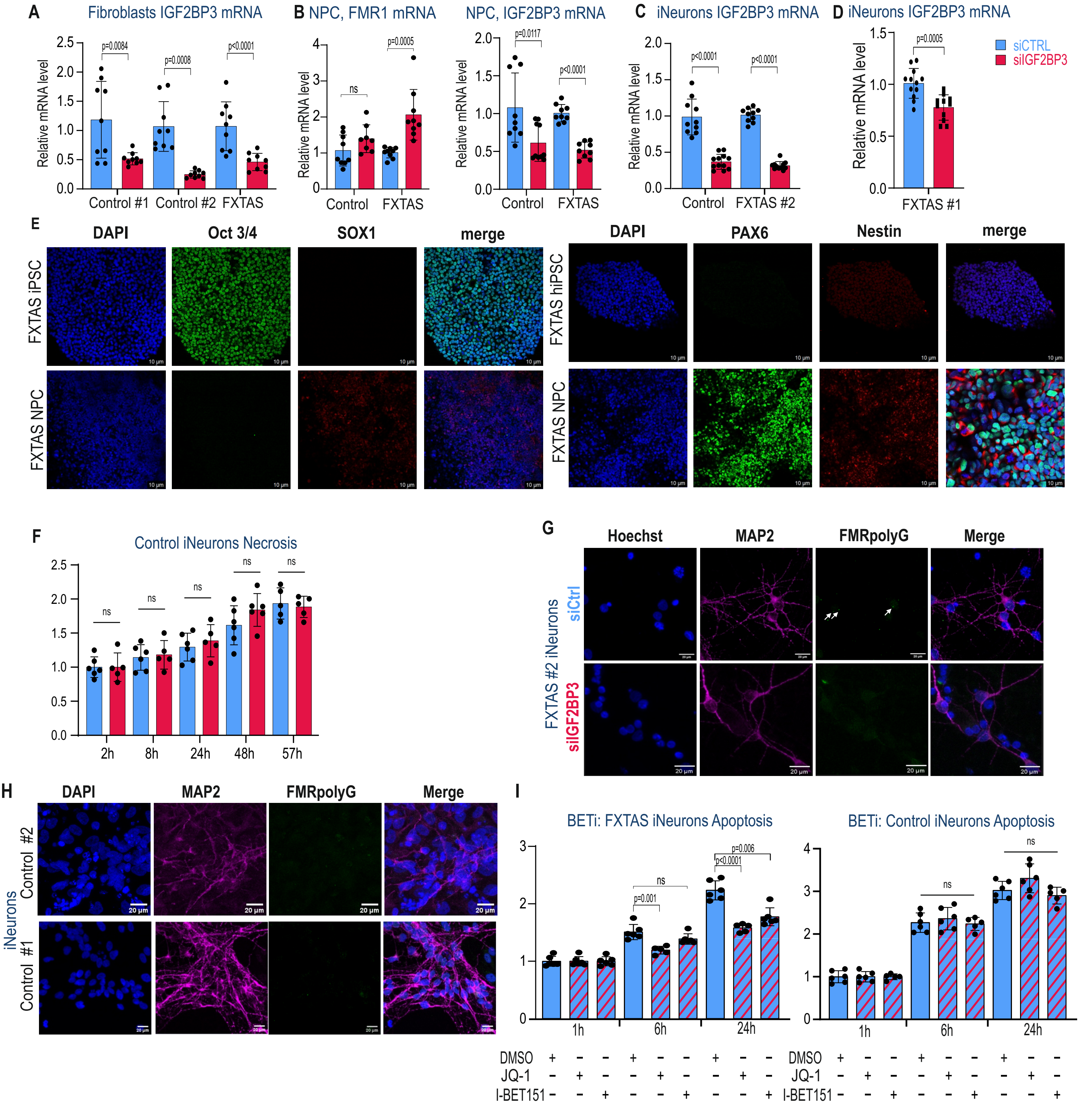

Figure S6. Data related to Figure 6. (A) *Related to Fig. 6A.* RT-qPCR of *IGF2BP3* levels in control and FXTAS fibroblasts with knocked down IGF2BP3 (magenta), relative to *GAPDH*. **(B)** RT-qPCR of *IGF2BP3* and *FMR1* levels in control and FXTAS neural progenitor cells (NPC) with knocked down IGF2BP3 (magenta), relative to *GAPDH*. (**C**) *Related to Fig. 6B.* RT-qPCR of *IGF2BP3* levels in control (29 CGG) and FXTAS (72 CGG) iPSC-derived neurons with knocked down IGF2BP3 (magenta). **(D)** *Related to Fig. 6D.* RT-qPCR of IGF2BP3 levels in FXTAS (83 CGG) iPSC-derived neurons with knocked down IGF2BP3, relative to GAPDH. **(E)** *Related to Fig.*6B-D Immunofluorescence against Oct 3/4, SOX1, PAX6 and Nestin in FXTAS iPSC and NPC, nuclei were stained with Hoechst 33342; scale bars, 10 μm. **(F)** *Related to Fig. 6C.* IGF2BP3 silencing does not impact necrosis rate in control (29CGG) iPSC-derived neurons. Necrosis was measured as fluorescence signals (relative fluorescence units). The graph presents relative mean values from N = 5 biologically independent samples treated with siCtrl (blue) or siIGF2BP3 (red), with Sds, normalized to fluorescence signals emitted at 1h. **(G)** *Related to Fig. 6D.* Immunofluorescence against FMRpolyG N-terminus was performed on neuronal cultures differentiated 21 days from control iPSC (left). **(H)** *Related to Fig. 6D.* Effect of IGF2BP3 silencing on FMRpolyG inclusions in FXTAS (72 CGG) iPSC-derived neurons. Immunofluorescence against FMRpolyG N-terminus (white arrows) was performed on neuronal cultures differentiated 21 days from FXTAS iPSC (left) on the background of IGF2BP3 silencing. Representative images were pseudo-colored and merged; green, FMRpolyG-positive inclusions; purple, neuronal marker MAP2; blue, nuclei stained with Hoechst 33342; scale bars, 20 μm. **(I)** BET inhibitors, JQ-1 and I-BET151 impact apoptosis rate in FXTAS (72 CGG) iPSC-derived neurons. Apoptosis was measured as relative luminescence units (RLU). The graph presents relative mean values from N = 6 biologically independent samples treated with DMSO (blue) or BET inhibitors (striped), with Sds, normalized to RLU emitted at 1h.

Supplementary Figure 7.

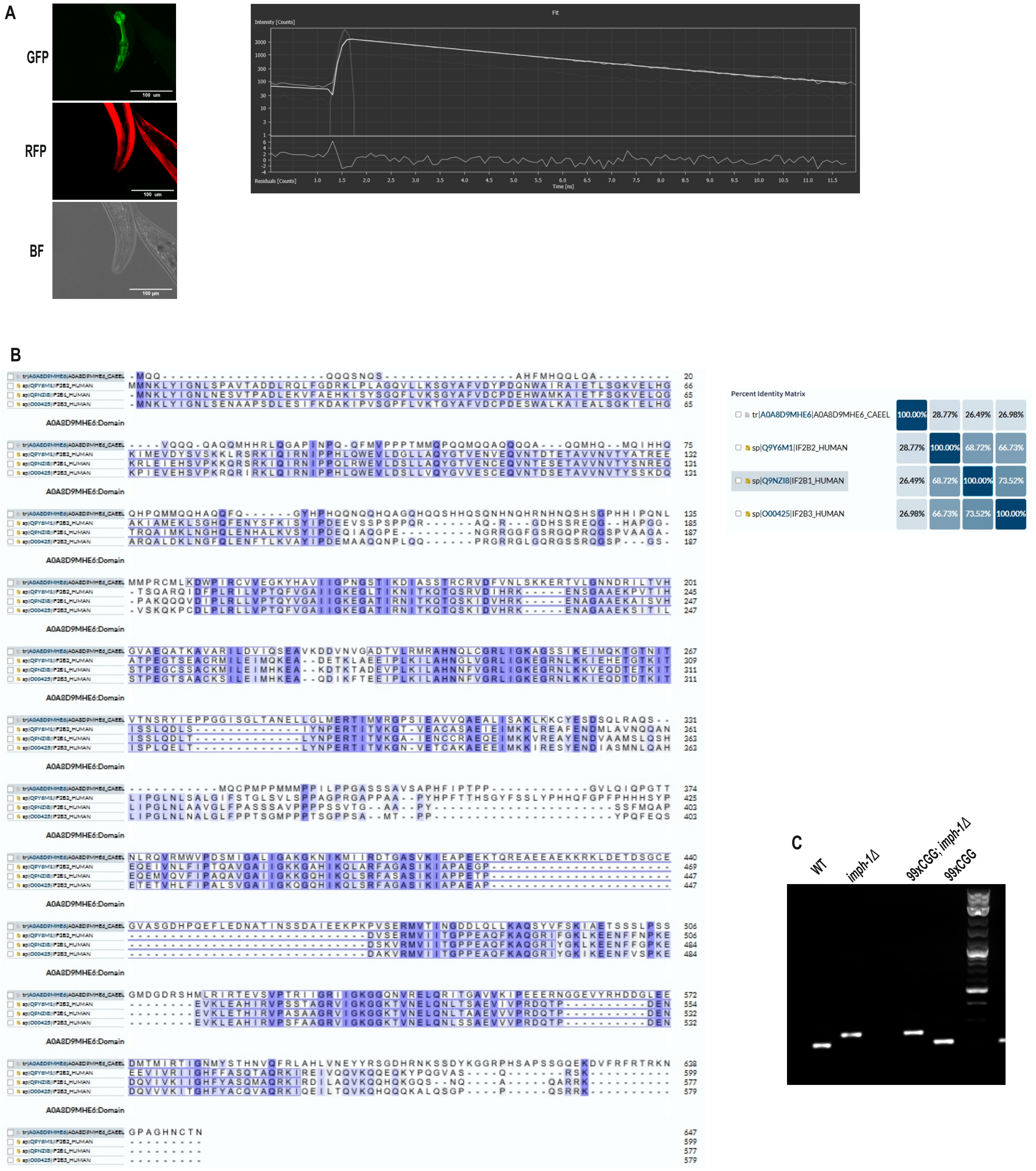

Figure S7. Data related to Figure 7. (A) *Related to Figure 7B*. Left: Microscopic images of FMRpolyG-GFP expression in pharynx *of C.elegans* 99xCGG model. Representative images were pseudo-colored: green, FMRpolyG-GFP; red, Red Fluorescence Protein; BF- bright-field; scale bars, 100 µm. Right: FLIM histogram of GFP signal in 99xCGG model. **(B)** Clustal 0 (1.2.4) multiple amino acid sequence alignment and percent of amino acid sequence of human IGF2BP paralogs and *C. elegans* imph-1. Sequence similarity among the paralogs is highlighted in blue. **(C)** Related to Figure 7B. Genotyping of *C.elegans* wild type (WT), 99xCGG, *imph-1Δ* and 99xCGG; *imph-1Δ* for the presence of a stop-in casette disrupting *imph-1* gene.

Primer Pair	Name	Sequence
F1/R1	F_5'UTRnoCGG	5'-GGCAGCGCTGGGCCTCGAGCGCCCGCA -3'
	R_5'UTRnoCGG	3'-AGGCCCAGCGCTGCCGCACGCCCCCT -5'
F2/R2	F_5UTR_FMRpolyG	5'-CGTTTAGTGAACCGTCTCAGTCAGGCGCTCAGCT -3'
	R_5UTR_FMRpolyG	5'-GAGTGTGAAGACCATCCTTGTAGAAAGCGCCATTGGA -3'
F3/R3	F_nLuc_FLAG	5'-TTACAAGGATGACGACGATAAGTAATTCTAGAGTCGGGGCGGC -3'
	R_nLuc_FLAG	5'-TCGTCATCCTTGTAATCTCCCGCCAGAATGCGTTCGCAC -3'
F4/R4	F_open_pNLv2	5'-ATGGTCTTCACACTCGAAGATTTCG -3'
	R_open_pNLv2	5'-ACGGTTCACTAAACGAGC -3'
F5/R5	F_Mut_16G_CA1 -TG	5'-GCGCCGCTGCTGGGGGGCGTGC -3'
	R_Mut_16G_CA1 -TG	5'-CTCCGTCACCGCCGCC -3'
F6/R6	F_GCrich	5'-CCGTGCTGCTCGCCCGGAG -3'
	R_GCrich	5'-GAAAGCGGAGCGGGTCTCGG -3'
F7	F_T7_GCrich	5'-TAATACGACTCACTATAGGGCCGTGCTGCTCGCCCGGAG -3'
F8/R8	F_5'UTR_FMR1_GFP	5'-GCAGCCCACCTCTCGGGG -3'
	R_5'UTR_FMR1_GFP	5'-CTTCGGGCATGGCGGACTTG -3'
F9/R9	F_hFMR1	5'-ATCCCAACAAACCTGCCACA -3'
	R_hFMR1	5’-ATGTGCTCGCTTTGAGGTGA -3’
F10/R10	F_hGAPDH	5'-GAGTCAACGGATTTGGTCGT -3'
	R_hGAPDH	5'-TTGATTTTGGAGGGATCTCG -3'
F11/R11	F_MALAT	5’-GACGGAGGTTGAGATGAAGC -3’
	R_MALAT	5’-ATTCGGGGCTCTGTAGTCCT -3’
F12/R12	F_GLO_401_imph -1	5'-ACAACAGAATCAAGGCGCTC -3'
	R_GLO_402_imph -1	5'-TGAGGCTGCATCATTGTTGG -3'
F13/R13	F_GLO_166_actin	5'-CAAGGAGTCATGGTCGGTATG -3'
	R_GLO_167_actin	5'-TCAATTGGGTACTTGAGGGTAAG -3'

Table S1. List of primers used in the study.

siRNA	Catalog number	Provider	Concentration
siIGF2BP1	sc-40694	SantaCruz Biotechnology	25 nM
siIGF2BP2	S20922	ThermoFisher Scientific	25 nM
siIGF2BP3	S20919	ThermoFisher Scientific	50 nM
siControl (single)	4390846	ThermoFisher Scientific	25 / 50 nM
siControl (pool)	D-001810-10-05	Dharmacon (Horizon)	25 nM

Table S2. List of siRNA used in the study.

References:

1. Nostrand, E. L. Van et al. Robust transcriptome-wide discovery of RNA-binding protein binding sites with enhanced CLIP (eCLIP). Nat. Methods 13, 508–514 (2016).
