# Supplementary material for: IGF2BPs directly regulate the noncanonical translation of toxic proteins from mutant FMR1 mRNA containing expanded CGG repeats
Source: Nat Commun. 2025 Dec 10;17:569. doi: 10.1038/s41467-025-67261-y (PMC12808120; doi:10.1038/s41467-025-67261-y)
Supplement: Supplementary file 2 — Description of Additional Supplementary Files [file 41467_2025_67261_MOESM2_ESM.pdf]

Description of Additional Supplementary Files

Supplementary Data 1. List of proteins binding to RNA baits.

Supplementary Movie 1. Wild type genotype motion.

Supplementary Movie 2. 99xCGG genotype motion.

Supplementary Movie 3. imph-1Δ genotype motion.

Supplementary Movie 4. 99xCGG; imph-1Δ genotype motion.
